# Supplementary material for: The interactome of intact mitochondria by cross-linking mass spectrometry provides evidence for coexisting respiratory supercomplexes
Source: Mol Cell Proteomics. 2017 Dec 8;17(2):216–32. doi: 10.1074/mcp.RA117.000470 (PMC5795388; doi:10.1074/mcp.RA117.000470)
Supplement: Supplemental Data [file supp_RA117.000470_133922_0_supp_23978_fzffwf.zip › spectra_annotation/mito_spectra_annotation/171-1-3-1-22-1.pdf]

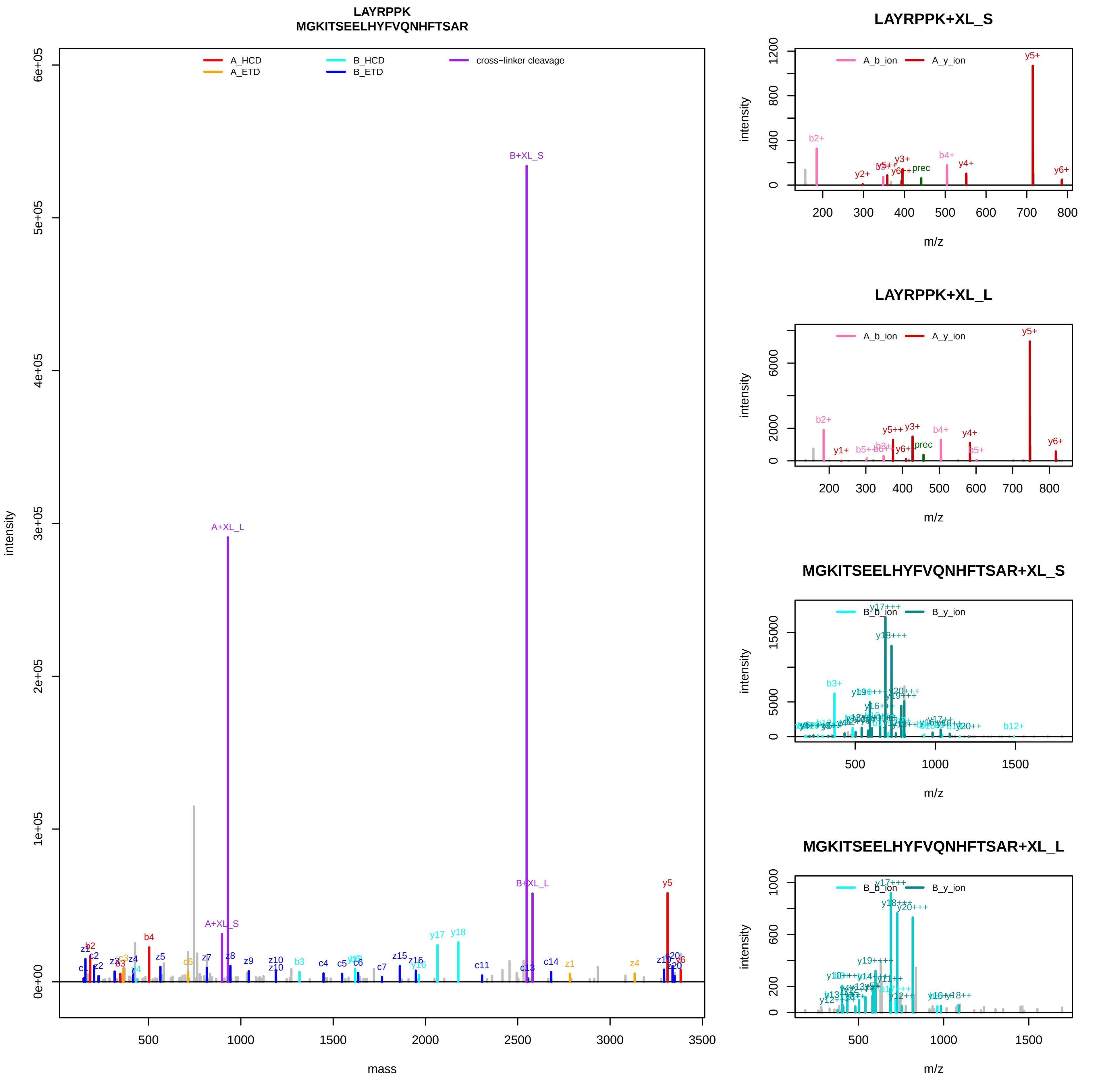
LAYRPPK
MGKITSEELHYFVQNHFTSAR
A_HCD
A_ETD
B_HCD
B_ETD
cross−linker cleavage
B+XL_S
A+XL_L
A+XL_S
B+XL_L
y5
y17
y18
b4
b2
c11
c13
c14
z15
z16
intensity
mass
LAYRPPK+XL_S
LAYRPPK+XL_L
MGKITSEELHYFVQNHFTSAR+XL_S
MGKITSEELHYFVQNHFTSAR+XL_L
A_b_ion
A_y_ion
B_b_ion
B_y_ion
y5+
b2+
b4+
y4+
y6+
y3+
prec
y17+++
y18+++
y20+++
m/z
